# The Impact of Novel BMPR1B Mutations on Litter Size in Short-Tailed Gobi Sheep and Larger-Tailed Ujimqin Sheep

**DOI:** 10.3390/vetsci11070297

**Published:** 2024-07-01

**Authors:** Yanyu Bai, Shenyuan Wang, Kaifeng Wu, Ming Zhang, Suhe Alatan, Ming Cang, Guifang Cao, Hai Jin, Changqing Li, Bin Tong

**Affiliations:** 1 The State Key Laboratory of Reproductive Regulation and Breeding of Grassland Livestock, School of Life Sciences, Inner Mongolia University, Hohhot 010020, China; baiyy_imu@163.com (Y.B.); cangming@imu.edu.cn (M.C.); 2 Inner Mongolia Key Laboratory of Biomanufacture, College of Life Sciences, Inner Mongolia Agriculture University, Hohhot 010020, China; wangshenyuan@imau.edu.cn; 3 College of Grassland, Resources and Environment, Inner Mongolia Agricultural University, Hohhot 010011, China; wukaifeng@126.com; 4 Inner Mongolia Mengyuan Sheep Breeding Company, Baotou 014016, China; meiligeng777@126.com; 5 East Ujimqin Hexig Animal Husbandry Development Company, Xilingol 026399, China; dwqyzc@163.com; 6 College of Veterinary Medicine, Inner Mongolia Agricultural University, Hohhot 010011, China; guifangcao@126.com; 7 Inner Mongolia Academy of Agricultural and Animal Husbandry Sciences, Hohhot 010031, China; jinhaicnm@vip.sina.com

**Keywords:** *BMPR1B*, litter size, Gobi short tail sheep, Ujimqin sheep, *T*/*Brachyury*

## Abstract

**Simple Summary:**

The *BMPR1B* and *T*/*Brachyury* genes are considered major genetic factors influencing sheep reproduction and tail bone number. In this study, we conducted an association analysis between nine mutations of the *BMPR1B* gene and the litter sizes of short-tailed Gobi sheep and larger-tailed Ujimqin sheep. We also compared the frequency of the favorable alleles of four mutations among Gobi short tail sheep, Ujimqin sheep, and Mongolia sheep. In addition, we confirmed that the frequency of tail-bone-number-related T alleles at position c.363G>T in the *T* gene was significantly higher in short-tailed Gobi sheep than larger-tailed Ujimqin sheep.

**Abstract:**

The significant deposition of tail fat in sheep has a profound impact on the economic benefits of animal husbandry. Furthermore, increasing the litter size is a crucial means of enhancing economic benefits. The *BMPR1B* and *T*/*Brachyury* genes are considered major functional genes that could affect sheep litter size and tail bone number, respectively. In this study, we employed direct sequencing to identify specific mutations of the *BMPR1B* gene in Gobi short tail sheep and carried out genotyping using MassARRAY technology for each variant of both the *BMPR1B* and *T* genes. Significant associations were demonstrated between the c.687G>A mutation of *BMPR1B* and the litter size in both the Gobi short tail sheep and Ujimqin sheep breeds. Meanwhile, the g.30058882_30058873GCAGATTAAAIndel mutation was significantly associated with the litter size in Gobi short tail sheep. These findings may provide valuable genetic markers for expanding sheep litter size. In addition, we also confirmed that the frequency of tail-bone-number-related T alleles was significantly higher in Gobi short tail sheep than in longer-tailed Ujimqin sheep.

## 1. Introduction

The Gobi short tail (GB) sheep is an excellent breed that is artificially bred from Mongolia sheep according to tail shape. It is mainly distributed in desert and semi-desert areas around Damao Banner, Dorbod Banner, and Urad Middle Banner, Inner Mongolia, China. GB sheep have the advantages of a small tail, cold and drought resistance, fast growth and development, and good vitality. In recent years, with improvements in people’s living standards and greater attention to cardiovascular diseases, the consumption of sheep tail fat has gradually decreased. Due to their small tail size, GB sheep tails contain relatively small fat deposits; this not only aligns with current public consumption preferences but also saves on breeding costs. The Ujimqin (UM) sheep is an ancient and primitive sheep breed that is mainly distributed in the grassland areas of northern China and southern Mongolia [1]. The primary distinction between the UM and GB breeds is the substantial size of the former’s tail and the fact that UM sheep are a homologous breed of Mongolia sheep (MG) [1,2].

The bone morphogenetic protein receptor 1B (*BMPR1B*) gene, belonging to the transforming growth factor-β (*TGF-β*) receptor family, is situated on ovine chromosome 6 and can directly or indirectly influence the differentiation of granulosa cells and the maturation of ovulatory follicles [3,4]. A renowned mutation, *FecB*, was discovered in Australian Merino Booroola sheep during the 1980s [5]. It involves a mutation from A to G at position 746 within the coding sequence (CDS) region, resulting in amino acid changes from glutamine to arginine [6]. However, in addition to its significant impact on Booroola sheep’s high yield, *FecB* remains effective in regulating reproduction in other sheep breeds, including Indian Garole sheep [7], Chinese Hu sheep [8], Iranian Kalehkoohi sheep [9], small-tailed Han sheep [10], and Mongolia sheep [11]. Although *FecB* has a significant effect on sheep litter size, the *FecB* mutation is rare in MG breeds [11].

Furthermore, in addition to the study of *FecB* in *BMPR1B*, many other single-nucleotide polymorphisms (SNPs) affecting litter size have been investigated. A novel SNP (C>A) was identified in exon 8 of *BMPR1B* in Mehraban sheep, and this mutation has a significant effect on litter size [12]. However, studies have also shown that mutations affecting sheep litter size are not limited solely to exon mutations; mutations within the 5’ untranslated region (UTR) and introns may also influence sheep reproductive traits [8]. Interestingly, in addition to SNPs that may affect litter size in sheep, large fragment insertion and deletion (indel) mutations may also play a role. One study reported the identification of a mutant genotype with a 10 bp deletion in Australian white sheep that was significantly associated with litter size [13]. In a previous study, *BMPR1B* was found to have breed-specific mutations in MG and UM, including exonic and six intronic mutations [14].

*Brachyury*, also known as T, is a member of the T-box family; its name derives from the Greek word “brakhus” [15]. In mice, this gene encodes a protein consisting of 436 amino acids and features a T-domain at the N-terminus that binds to palindromic sequences in the genome [16]. Research has shown that mesodermal cells continue to express *Brachyury* upon the conclusion of gastrulation [17]. In mice, *Brachyury*/*T* is indispensable for early embryonic development, playing a critical role in mesoderm formation and tail structure development [18]. The knockout of this gene in mice results in a significantly shortened tail, often accompanied by a slight curvature [19]. Similarly, in sheep, a G363T mutation in this gene leads to a reduction in the number of tail bones [20].

The aim of this study was to explore breed-specific mutations in GB and UM sheep, analyze the genetic diversity and its associations with litter size, and compare the frequencies of favorable alleles of known mutations in different sheep breeds. Additionally, this study compares the frequency of tail-bone-number-related T alleles in GB and UM sheep.

## 2. Materials and Methods

### 2.1. Animals and Samples

The 231 Gobi short tail sheep used in this study were raised on the Dorbod Banner Sheep Farm, while the 153 Ujimqin sheep used in this study originated from a Ujimqin sheep-breeding farm in East Ujimqin under similar conditions, with free access to food and water and natural lighting. Blood was collected from the jugular vein (10 mL/sample) using EDTA-K2 anticoagulation tubes, and then stored at −20 °C. All animal care and experiments were conducted according to the Administration of Affairs Concerning Experimental Animals China. The research protocol was approved on 15 May 2015 by the Institutional Animal Care and Use Ethics Committee of Inner Mongolia University, with a permit for conducting animal experiments (number IMU-2015-03).

### 2.2. DNA Extraction and Sequencing

DNA was extracted from 384 blood samples using the Tiangen Blood/Cell/Tissue Genomic DNA Extraction Kit (catalog number: DP304; Tiangen Biotechnology Co., Ltd., Beijing, China), following the instructions provided by the manufacturer. To prevent degradation, agarose gel electrophoresis and UV spectrophotometry were used to assess the quality and concentration of DNA after storage at −20 °C.

PCR amplification of 13 primer pairs was carried out with Primer Premier 5.0 to amplify the promoter and exon regions of Gobi short tail sheep *BMPR1B* (Oar_rambouillet_v2.0, NCBI reference sequence: NC_056059.1), from six randomly selected samples. These PCR amplifications were performed with 2 μL of the prepared DNA as a template in a final volume of 50 μL containing 1 mM of each primer, 25 mL of Ex Taq DNA polymerase (Takara, Dalian, China), and 21 mL of ddH_2_O. The PCR conditions were as follows: 94 °C for 5 min, 35 cycles of 94 °C for 30 s, annealing for 30 s, 72 °C for 1 min 10 s, and a final extension step at 72 °C for 10 min. The annealing temperatures for each fragment are shown in Table 1. The PCR products were analyzed via 3.0% agarose gel electrophoresis to determine the DNA sequencing quality and quantity. The products were sequenced by the Beijing Genomics Institute (BGI, Beijing, China).

### 2.3. SNP Genotyping Using iPLEX MassARRAY

Five novel variants were genotyped with the MassARRAY^®^ SNP genotyping system (Agena Bioscience, San Diego, CA, USA) in the 231Gobi short tail sheep and 153 Ujimqin sheep. The PCR and extension primers of *BMPR1B* were designed from sequences containing each target mutation and ~100 upstream and downstream bases via the Assay Design Suite v3.0 (http://agenabio.com/assay-design-suite-20-software, accessed on 15 December 2023), using the default settings (Table S1). The genotype of each allele was analyzed using the Sequenom MassARRAY iPLEX platform. The resulting data were analyzed using MassARRAY Typer 4.0 Analyzer software (Agena Bioscience, San Diego, CA, USA) [21].

### 2.4. Statistical Analyses

The allele frequency, heterozygosity, polymorphism information content, and Hardy–Weinberg equilibria were calculated using Excel. Linkage disequilibrium (LD), including D’ and *r*^2^, was assessed using HAPLOVIEW v. 4.2 [22]. Using a *χ* ^2^ test, the allelic frequency within each mutation was analyzed. A two-way chi-squared test was used to examine the genetic influences of each SNP in the GB and UM alleles on litter size [14,23,24,25]. Their associations and effects could not be accurately evaluated when the number of sheep with a certain genotype was lower than ten. All results are reported as the mean ± SEM values.

## 3. Results

### 3.1. Variant Discovery in the BMPR1B Gene of Gobi Short Tail Sheep

**Figure 1:**
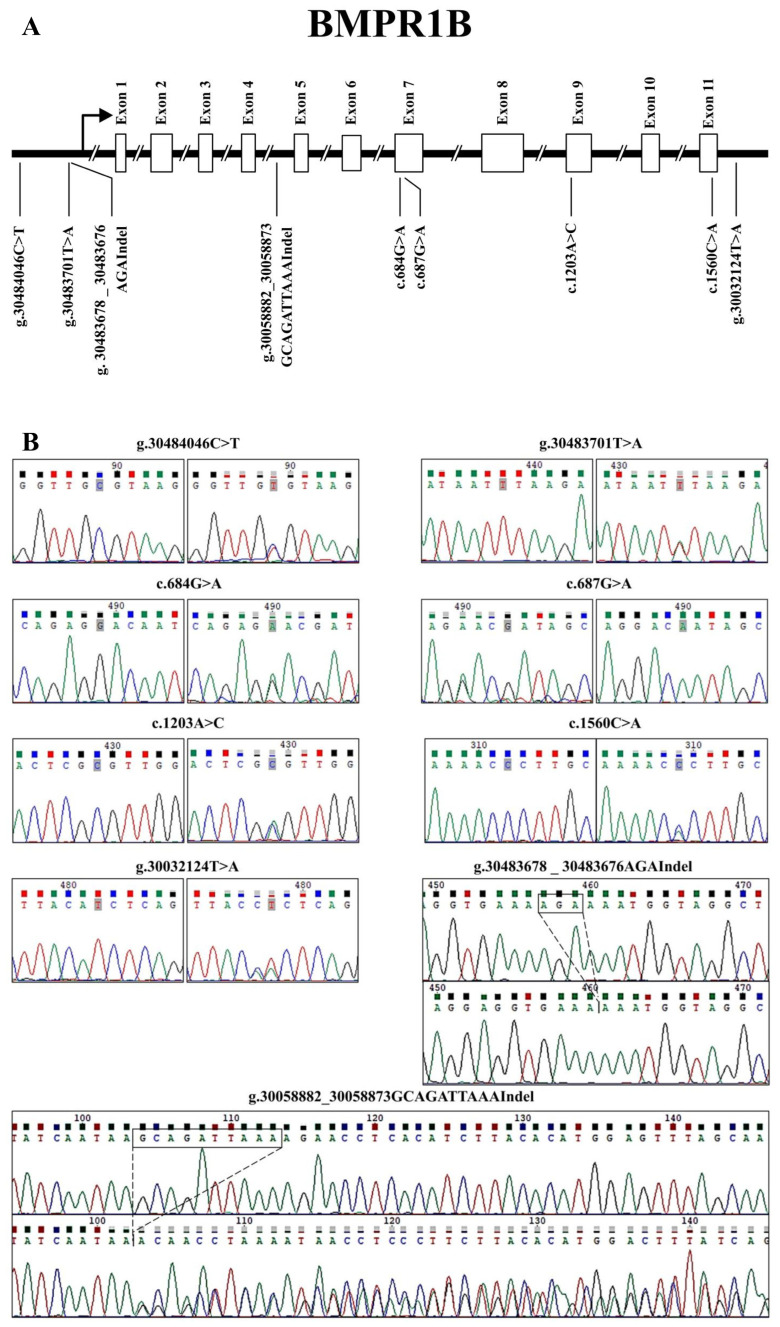
The identification of nine ovine *BMPR1B* variants. (**A**) The physical locations of each of the nine variants identified in this study are shown. (**B**) The nucleotide substitutions of the nine *BMPR1B* variants are shown. The variants are according to chromosome 6 in Oar_rambouillet_v2.0 (GenBank accession: NC_056059.1).

A sequence analysis revealed nine *BMPR1B* variants. The mutations located in exons 7, 9, and 11 of *BMPR1B*, namely c.684G>A, c.687G>A, c.1203A>C, and c.1560C>A, respectively, were synonymous to the mutations previously identified in Mongolia sheep by our laboratory [13]. In this study, we discovered five novel mutations within *BMPR1B* comprising three SNP and two indel mutations. Specifically, two single-nucleotide mutations (g.30484046C>T and g.30483701T>A) were found in the promoter, and one single-nucleotide mutation (g.30032124T>A) was found in the 3’ UTR of *BMPR1B*. Additionally, mutations c.30483678_30483676AGAIndel and g.30058882_30058873GCAGATTAAAIndel were located in the promoter and intron 4 regions of *BMPR1B*, respectively (Figure 1).

### 3.2. Genetic Diversity Analysis

For each variant we identified, the frequencies of the two alleles and three genotypes in the GB and UM sheep are listed, along with the genetic indices (H_o_, H_e_, n_e_, and PIC) (Table S2). The frequencies of the A and T alleles and the A allele in the c.684G>A, g.30484046C>T, and g.30032124T>A SNPs showed a relatively low distribution in each sheep breed. Among them, in all experimental samples, with the exception of c.684G>A and g.30484046C>T, which showed low levels of polymorphism in both the GB and UM sheep (PIC < 0.25), the other mutations showed moderate polymorphism in both breeds (0.25 < PIC < 0.5), and there was no highly polymorphic site (PIC > 0.5).

### 3.3. Linkage Disequilibrium Analysis of Novel Variants in BMPR1B

**Figure 2:**
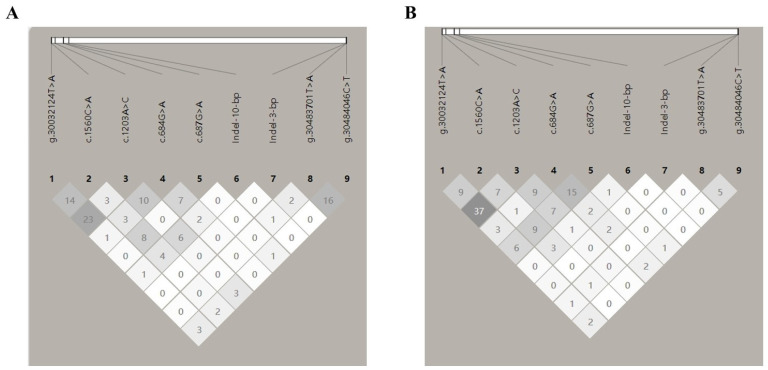
Linkage disequilibrium (LD) estimated among *BMPR1B* variations in the Gobi short tail sheep and Ujimqin sheep populations. Numbers represent *r*^2^ × 100. (**A**) Gobi short tail sheep. (**B**) Ujimqin sheep. Indel-10-bp: g.30058882_30058873GCAGATTAAAIndel; Indel-3-bp: g.30483678_30483676AGAIndel. The lines refer to the relative positions of the loci on the chromosome. Different shades of gray indicate the degree of linkage disequilibrium between the two loci. The darker shows the higher linkage disequilibrium between the two loci.

To identify the linkage relationships among the nine SNPs, the D’ and *r^2^* were estimated for the GB and UM breeds. The resulting *r*^2^ values indicate that all the sites presented a low linkage disequilibrium for these experimental sheep populations (Figure 2, Tables S3 and S4).

### 3.4. Associations between Novel Variants and Litter Size

Due to the influence of the *FecB* mutation on sheep litter size, we tested for *FecB* in GB and UM ewes at first, and there were no *FecB* mutations in our experimental animals (Table S2). On this basis, the effects of the nine SNPs on litter size were analyzed in the GB and UM specimens. The results show that the litter size for the GG genotype of c.687G>A was significantly higher (*p* < 0.05) than those for the GA and AA genotypes in the GB sheep, and the litter sizes for the GG and GA genotypes were significantly higher (*p* < 0.05) than that of the AA genotype in the UM sheep, (Table 2 and Table 3 and Figure S2). In addition, for g.30058882_30058873GCAGATTAAAIndel, the litter size of the GB sheep with the GCAGATTAAA.DEL genotype was significantly higher (*p* < 0.05) than that of the sheep with the GCAGATTAAA.GCAGATTAAA and DEL.DEL genotypes (Table 2 and Figure S2).

### 3.5. Comparison of Allele Frequency of Nine BMPR1B Variants

**Figure 3:**
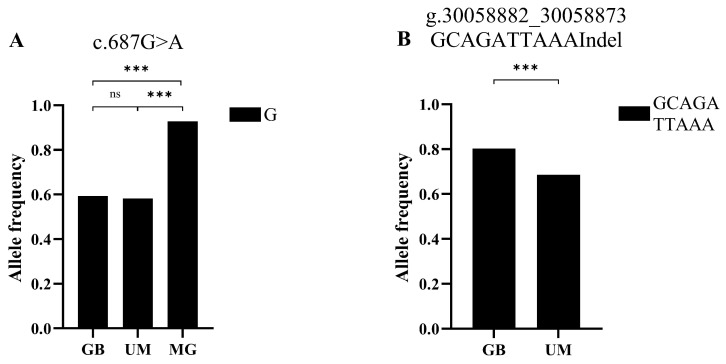
Distribution of allele frequencies of two *BMPR1B* mutations in different sheep breeds. (**A**) G allelic frequencies of c.684G>A in *BMPR1B* in Gobi short tail sheep, Ujimqin sheep, and Mongolia sheep. (**B**) CGAGATTAAA allelic frequencies of g.30058882_30058873 CGAGATTAAAIndel in *BMPR1B* in Gobi short tail sheep and Ujimqin sheep. GB: Gobi short tail sheep; UM: Ujimqin sheep; MG: Mongolia sheep. n.s.: non-significant; ***: *p* < 0.001.

The allelic frequencies of the four known mutations [14] were compared among the GB, UM, and MG sheep (using previously published MG data from our laboratory), including the litter size-associated G allele of c.687G>A and the GCAGATTAAA allele of g.30058882_30058873GCAGATTAAAIndel in *BMPR1B,* which were compared among the GB, UM, and GM, and GB and UM breeds, respectively. The frequencies of known mutations in the GB, UM, and MG breeds and those of ones newly discovered in GB and UM sheep were compared. The results indicate that the frequency of the litter size-associated G allele of c.687G>A was significantly higher in the GM sheep than in the GB and UM populations, while the frequency of the GCAGATTAAA allele of g.30058882_30058873GCAGATTAAAIndel was significantly higher in the GB sheep than in the UM sheep (Figure 3). In addition, there were significant or significantly higher differences between the GB and UM sheep at c.684G>A and c.1203A>C among the known mutations, as well as significant or significantly higher differences between the GB and MG sheep at c.684G>A, c.687G>A, and c.1203A>G (Figure S1). Among the newly discovered mutations, with the exception of g.30484046C>T and g.30032124T>A, where no difference in allelic frequencies was observed between the GB and UM sheep, all the mutations showed significantly higher differences in the allelic frequencies between the GB and UM breeds (Figure S1).

### 3.6. Comparison of T Allele Frequencies

**Figure 4:**
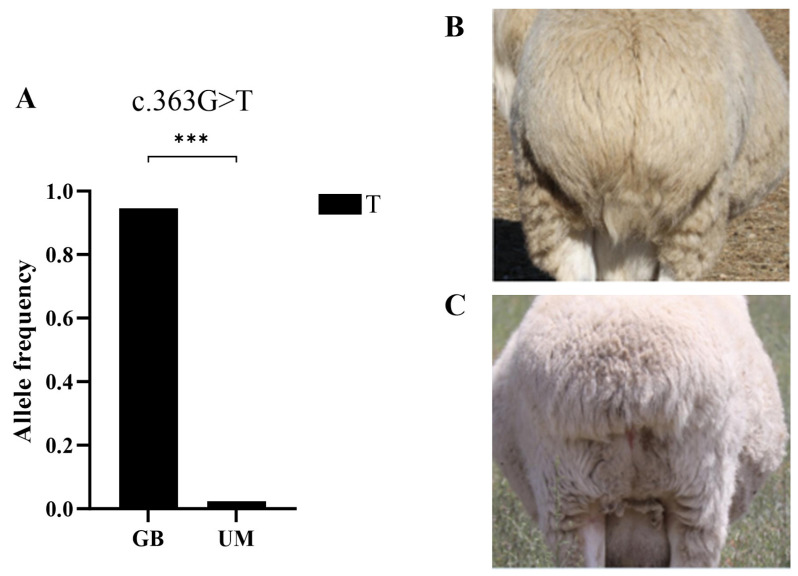
Distribution of allele frequencies of *T*/*Brachyury* mutation in two sheep breeds. (**A**) T allelic frequencies of c.363G>T in *T*/*Brachyury* in Gobi short tail sheep and Ujimqin sheep. (**B**) Tail of Ujimqin sheep. (**C**) Tail of Gobi short tail sheep. GB: Gobi short tail sheep; and UM: Ujimqin sheep. n.s.: non-significant; ***: *p* < 0.001.

To better distinguish between GB and UM sheep, comparisons of allelic frequencies were conducted for the *T*/*Brachyury* gene, which influences the number of tail bones. The results indicate that the frequency of the T allele was significantly higher in the GB sheep than in the UM sheep (Figure 4 and Table S2).

## 4. Discussion

Since the identification of the *FecB* gene, it has been recognized that mutations within the *BMPR1B* gene may affect sheep prolificacy [5]. For instance, the g.29362047T>C and g.29427689G>A SNPs have been shown to influence litter size in Merino sheep [12]. Additionally, a synonymous mutation at position T37K was found to be associated with litter size in Hu sheep [8], while the C864T mutation has been observed to impact litter size in small-tailed Han ewes [26]. This study identified nine mutations within the GB population. Although four of these mutations were detected in the exonic region of *BMPR1B*, the degeneracy of codons prevented alterations in the encoded amino acids at these positions. Nonetheless, Karimian et al. [27] show that mutations resulted in a decrease in the minimum free energy (MFE) of the mRNA, thereby imparting greater stability to *MTHFR* mRNA, potentially altering *MTHFR* gene expression. In addition, some synonymous mutations have been shown to lead to decreased mRNA stability and translation efficiency, significantly altering the dopamine-induced upregulation of *DRD2* expression [28]. These studies have demonstrated the impact of synonymous mutations at the RNA and protein levels. Data on alterations in RNA secondary structure and MFE values resulting from the exonic mutations identified in this study have been previously published by our laboratory [14].

Of particular interest, in Chinese Australian White sheep, insertions and deletions of 10 bp and 12 bp within *BMPR1B* have been found to alter RNA splicing, with the 10 bp indel being significantly correlated with prolificacy [13]. Additionally, a different study discovered that a close linkage disequilibrium between a 90 bp indel mutation and g.746A>G is significantly associated with prolificacy in Hu sheep [29]. These findings suggest that indel mutations may also influence RNA splicing, stability, and, potentially, sheep prolificacy. In our study, indel mutations were identified as novel mutations, such as g.30058882_30058873GCAGATTAAAIndel, located in intron 4 and exhibiting moderate polymorphism in both GB and UM sheep. However, experimental validation is necessary to confirm the hypothesis that altering *BMPR1B* mRNA stability and secondary structure affects sheep prolificacy.

Despite the significant regulatory role of the *FecB* mutation in sheep reproduction, many sheep breeds lack *FecB* and present alterations in their litter size that are not attributable to *FecB*. Previous studies in our laboratory have reported associations between mutations and litter size in *BMP15*. Specifically, g.50985975G>A and c.755T>C were found to influence litter size in MG sheep, while g.50988478C>A and g.50987863G>A were found to influence litter size in UM sheep in samples lacking *FecB* mutations [30]. Sheep prolificacy is directly associated with the ovulation rate, which is regulated by follicular development and oocyte maturation—a highly complex process [31]. For example, the joint regulation of prolificacy by *FecX^L^* and *FecL* has been observed in Lacaune sheep [32], while *FecB* and *FecX^G^* together influence prolificacy in small-tailed Han sheep, indicating diverse ovulation mechanisms across different sheep breeds [10]. The findings of this study present new opportunities for exploring the correlation between *BMPR1B* and sheep prolificacy, as well as investigating the function of this gene. Therefore, future research should confirm the relationship between variations in GB and UM breeds and prolificacy in larger sheep populations.

In addition, as the standard of living improves, there is a gradual decline in people’s preference for sheep tail fat. However, the meat quality and nutritional value of Mongolia sheep breeds continue to be highly valued, leading to the increasing visibility of the GB variety among consumers. Indeed, studies have shown an association between the shortness of the tail and fat deposition [33], and the tails size is a crucial indicator distinguishing GB sheep. Previous studies reported that mutations in the *T* gene in MG sheep can lead to variations in the number of tail bones [20]. Meanwhile, research has shown that heterozygous mice with null mutations in the *T* gene exhibit curved, short tails or tails of half the normal length. Homozygous mutant mice display spinal cord defects, leading to a failure to develop normal structures during the late embryonic stages, and resulting in death shortly after birth or severe defects [34]. Similar phenomena have been observed in *T* gene studies in mice and zebrafish [35,36]. Therefore, after collecting well-defined breeds from various core breeding facilities, the samples in this study were further validated by examining the *T* gene, which influences tail bones. The results indicate that among the 231 GB and 153 UM sheep in our study sample, the frequency of the tail-bone-number-related T allele was significantly higher in the GB sheep than in the UM sheep.

## 5. Conclusions

In this study, the association results suggest that the known c.687G>A mutation and the novel g.30058882_30058873GCAGATTAAAIndel mutation within *BMPR1B* could influence the litter size of Gobi short tail sheep and Ujimqin sheep. In addition, we have confirmed that the frequency of the tail-bone-number-related T allele was significantly higher in short-tailed Gobi sheep. These data provide potential genetic markers for sheep breeding and offer new potential avenues for investigating *BMPR1B* polymorphism.

## Figures and Tables

**Table 1:** PCR primers used for sequencing *BMPR1B*.

Primer	Primer Sequence (5′–3′)	Target Region	Annealing Temperature Tm (°C)	Product Size (bp)
BMPR1B-P1	F: GGAAGGTAATTTAGGAGCAC	Promoter	54	782 (−1575 ~ −793 bp upstream of promoter)
	R: TCAGGAAACTTGGGGATA			
BMPR1B-P2	F: CCAGAACGCAGGTTAGTC	Promoter	54	1060 (−1366 ~ −306 bp upstream of promoter)
	R: CAGATGCTTTCTCGGTCA			
BMPR1B-P3	F: CCTTGATGAGTTTCAGTCCA	Promoter	60	896 (−446 bp upstream of promoter + 450 bp 5‘ UTR)
	R: TCGGTGCGTCCCTCCTTC			
BMPR1B-1	F: CGATTCCCAAAGAATTACCA	Exon 1	56	808 (472 bp 5‘ UTR + 73 bp exon 1 + 263 bp intron 1)
	R: TTCCAAACTCGACTGCACAT			
BMPR1B-2	F: AAGACTAGTAGAAGTTTTAGGGC	Exon 2	60	1044 (451 bp intron1 + 160 bp exon 2 + 433 bp intron 2)
	R: AGGGTTCTTTATGACTAGCAC			
BMPR1B-3/4	F: TTACCCTGGACATGCTCAC	Exons 3/4	60	1000 (101 bp intron 2 + 103 bp exon 3 + 512 bp intron 3 + 103 bp exon 4 + 181 bp intron 4)
	R: ATTTTCCCAGTAACTCCCT			
BMPR1B-5	F: TGTGTCAAACCTGTTCCTTCC	Exon 5	60	659 (260 bp intron 4 + 97 bp exon 5 + 302 bp intron 5)
	R: CAACACAGTCATTTCTTGCTTTG			
BMPR1B-6	F: CACTCCAGTATGTTTGCCT	Exon 6	55	910 (412 bp intron 5 + 139 bp exon 6 + 359 bp intron 6)
	R: CACTCCAGTATGTTTGCCT			
BMPR1B-7	F: GAGTATCTAGCGTCCCACA	Exon 7	58	1059 (506 bp intron 6 + 193 bp exon 7 + 360 bp intron 7)
	R: CCTGCAGATAAAATTCCCAT			
BMPR1B-8	F: CTCTCATAAGCACAAGCAG	Exon 8	55	746 (143 bp intron 7 + 298 bp exon 8 + 305 bp intron 8)
	R: TTCTGAGCACACAATCCCA			
BMPR1B-9	F: TCCTTTCCCTTCTTACTTGTT	Exon 9	58	1098 (422 bp intron 8 + 176 bp exon 9 + 500 bp intron 9)
	R: ATTTATGTTTTCAAGCTCGTT			
BMPR1B-10	F: AATAATGTTTCCGTGTGCTT	Exon 10	53	752 (224 bp intron 9 + 131 bp exon 10 + 397 bp intron 10)
	R: AATTCTTCAGATGCCTACCTC			
BMPR1B-11	F: ACACTCTTCTACTATCAGCAA	Exon 11	55	658 (253 bp intron 10 + 126 bp intron 11 + 279 bp 3’ UTR)
	R: AAGGCAATCCCAAAATACCG			

Note. F: forward primer; R: reverse primer; UTR: untranslated region.

**Table 2:** The effects of the genotypes of the nine *BMPR1B* variants on litter size in the Gobi short tail sheep population.

Variant	Genotype	Number	Litter Size
c.684G>A	GG	187	1.03 ± 0.10
	GA	43	1.09 ± 0.12
c.687G>A	GG	84	1.38 ± 0.17 ^a^
	GA	106	1.28 ± 0.17 ^b^
	AA	41	1.30 ± 0.18 ^b^
c.1203A>C	AA	54	1.36 ± 0.18
	AC	120	1.33 ± 0.17
	CC	57	1.28 ± 0.17
c.1560C>A	CC	84	1.34 ± 0.17
	CA	107	1.33 ± 0.17
	AA	40	1.30 ± 0.17
g.30484046C>T	CC	180	1.37 ± 0.15
	CT	47	1.47 ± 0.16
g.30483701T>A	TT	99	1.33 ± 0.17
	TA	100	1.29 ± 0.17
	AA	32	1.35 ± 0.17
g.30032124T>A	TT	155	1.37 ± 0.16
	TA	66	1.31 ± 0.17
	AA	10	1.28 ± 0.21
Indel-3-bp	AGA.AGA	122	1.38 ± 0.16
	AGA.DEL	86	1.36 ± 0.17
	DEL.DEL	23	1.29 ± 0.18
Indel-10-bp	GCAGATTAAA. GCAGATTAAA	152	1.31 ± 0.16 ^a^
	GCAGATTAAA.DEL	67	1.46 ± 0.17 ^b^
	DEL.DEL	12	1.20 ± 0.20 ^a^

Note: ^a^, ^b^: *p* < 0.05. Indel-10-bp: g.30058882_30058873GCAGATTAAAIndel; Indel-3-bp: g.30483678_30483676AGAIndel.

**Table 3:** The effects of the genotypes of the nine *BMPR1B* variants on litter size in the Ujimqin sheep population.

Variant	Genotype	Number	Litter Size
c.684G>A	GG	107	1.04 ± 0.17
	GA	39	1.02 ± 0.18
c.687G>A	GG	52	1.15 ± 0.19 ^b^
	GA	74	1.11 ± 0.18 ^b^
	AA	27	0.80 ± 0.19 ^a^
c.1203A>C	AA	18	1.19 ± 0.19
	AC	57	0.90 ± 0.19
	CC	78	0.97 ± 0.19
c.1560C>A	CC	44	0.95 ± 0.17
	CA	79	0.99 ± 0.18
	AA	30	1.11 ± 0.19
g.30484046C>T	CC	126	1.15 ± 0.10
	CT	26	1.20 ± 0.13
g.30483701T>A	TT	45	1.10 ± 0.19
	TA	69	1.00 ± 0.18
	AA	39	0.95 ± 0.17
g.30032124T>A	TT	51	1.01 ± 0.17
	TA	68	1.17 ± 0.16
	AA	34	0.87 ± 0.28
Indel-3-bp	AGA.AGA	114	1.01 ± 0.18
	AGA.DEL	35	1.03 ± 0.18
Indel-10-bp	GCAGATTAAA. GCAGATTAAA	75	1.00 ± 0.17
	GCAGATTAAA.DEL	60	1.10 ± 0.18
	DEL.DEL	18	1.01 ± 0.20

Note: ^a^, ^b^: *p* < 0.05. Indel-10-bp: g.30058882_30058873GCAGATTAAAIndel; Indel-3-bp: g.30483678_30483676AGAIndel.

## Data Availability

Data are contained within the article and supplementary materials.

## Supplementary Materials

The following supporting information can be downloaded at https://www.mdpi.com/article/10.3390/vetsci11070297/s1: Figure S1: Distribution of allele frequencies of seven *BMPR1B* mutations in different sheep breeds; Figure S2: Litter size distribution of variety of genotypes in different sheep breeds; Table S1: MassARRAY primers used for genotyping ten variants in *BMPR1B* and one variant in *T* gene; Table S2: Genotypic allelic frequencies and diversity parameters of eleven SNPs in Gobi short tail sheep and Ujimqin sheep populations; Table S3: Linkage disequilibrium as measured using D’ and *r^2^* among variants in Gobi short tail sheep population; Table S4. Linkage disequilibrium as measured using D’ and *r^2^* among variants in Ujimqin sheep population.
