# Supplementary material for: The Impact of Novel BMPR1B Mutations on Litter Size in Short-Tailed Gobi Sheep and Larger-Tailed Ujimqin Sheep
Source: Vet Sci. 2024 Jul 1;11(7):297. doi: 10.3390/vetsci11070297 (PMC11281403; doi:10.3390/vetsci11070297)
Supplement: Supplementary file 1 [file vetsci-11-00297-s001.zip › Figure S1 Distribution of allele frequencies of seven BMPR1B mutations in different sheep breeds.pdf]

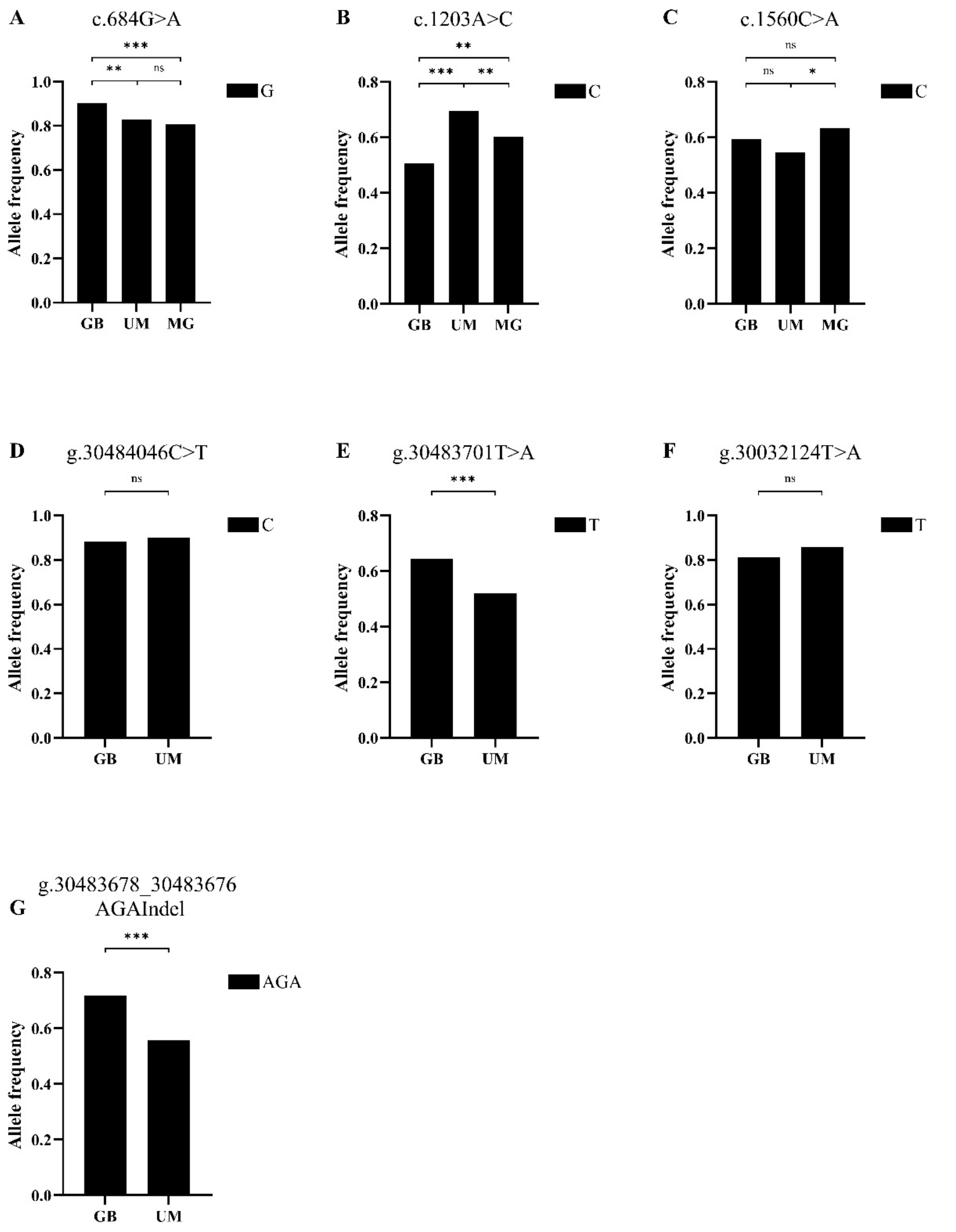

Figure S1. Distribution of allele frequencies of seven *BMPR1B* mutations in different sheep breeds. (A) The G allelic frequencies of c.684G>A in *BMPR1B* in Gobi short tail sheep, Ujimqin sheep and Mongolia sheep. (B) The C allelic frequencies of c.1203A>C in *BMPR1B* in Gobi short tail sheep, Ujimqin sheep and Mongolia sheep. (C) The C allelic frequencies of c.1560C>A in *BMPR1B* in Gobi short tail sheep, Ujimqin sheep and Mongolia sheep. (D) The C allelic frequencies of g.30484046C>T in *BMPR1B* in Gobi short tail sheep and Ujimqin sheep. (E) The T allelic frequencies of g.30483701T>A in *BMPR1B* in Gobi short tail sheep and Ujimqin sheep. (F) The T allelic frequencies of g.30032124T>A in *BMPR1B* in Gobi short tail sheep and Ujimqin sheep. (G) The AGA allelic frequencies of g.30483678_30483676AGAIndel in BMPR1B in Gobi short tail sheep and Ujimqin sheep. GB: Gobi short tail sheep, UM: Ujimqin sheep, MG: Mongolia sheep. n.s.: Non-significant; *: $p < 0.05$; **, ***: $p < 0.01$.
