# Supplementary material for: The Impact of Novel BMPR1B Mutations on Litter Size in Short-Tailed Gobi Sheep and Larger-Tailed Ujimqin Sheep
Source: Vet Sci. 2024 Jul 1;11(7):297. doi: 10.3390/vetsci11070297 (PMC11281403; doi:10.3390/vetsci11070297)
Supplement: Supplementary file 1 [file vetsci-11-00297-s001.zip › Figure S2 Distribution of litter size of variety genotypes in different sheep breeds. .pdf]

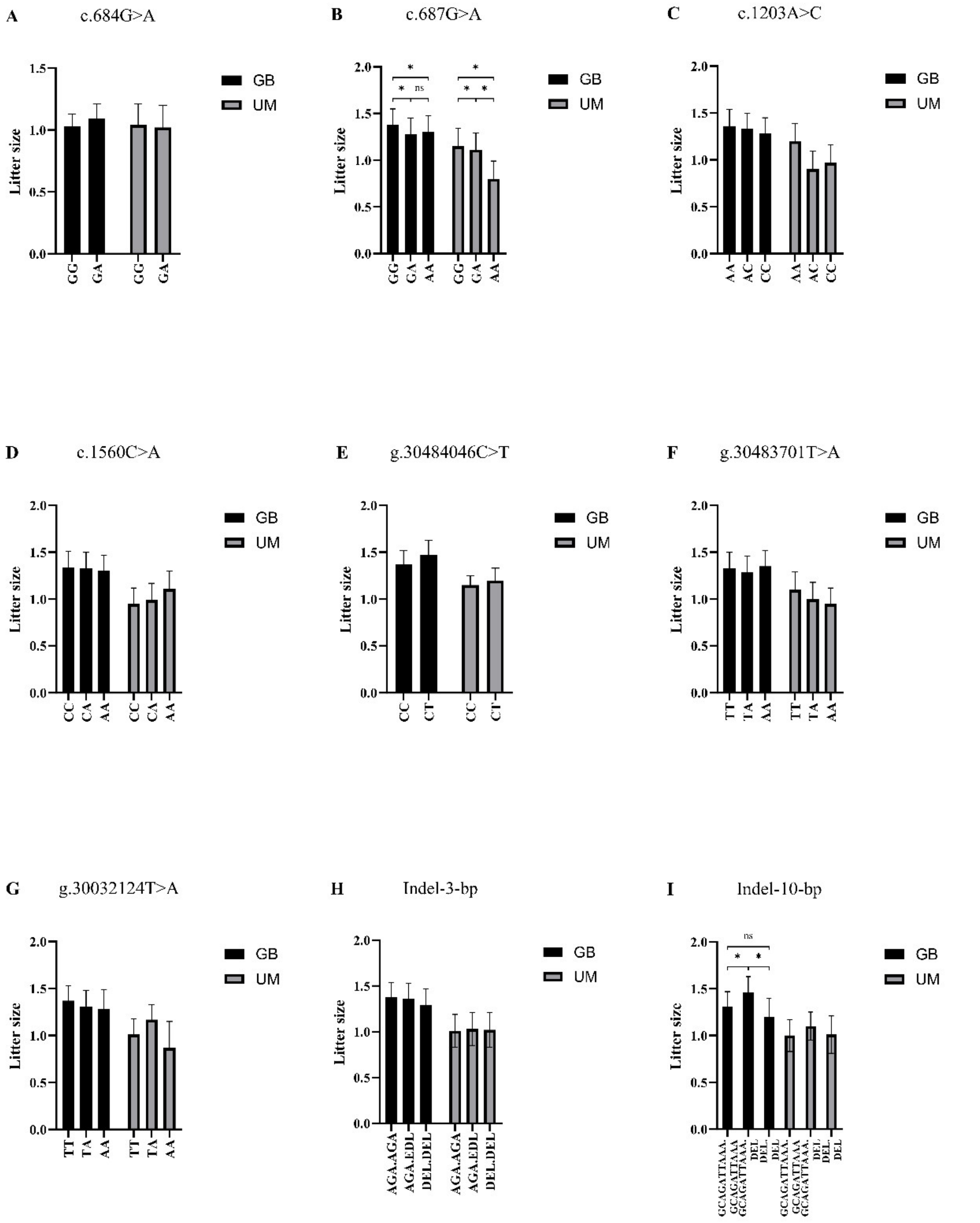


**Figure S2.** Distribution of litter size of variety genotypes in different sheep breeds. (A) The litter size of c.684G>A in Gobi short tail sheep and Ujimqin sheep. (B) The litter size of c.687G>A in Gobi short tail sheep and Ujimqin sheep. (C) The litter size of c.1203A>C in Gobi short tail sheep and Ujimqin sheep. (D) The litter size of c.1560C>A in Gobi short tail sheep and Ujimqin sheep. (E) The litter size of g.30484046C>T in Gobi short tail sheep and Ujimqin sheep. (F) The litter size of g.30483701T>A in Gobi short tail sheep and Ujimqin sheep. (G) The litter size of g.30032124T>A in Gobi short tail sheep and Ujimqin sheep. (H) The litter size of Indel-3-bp in Gobi short tail sheep and Ujimqin sheep. (I) The litter size of Indel-3-bp in Gobi short tail sheep and Ujimqin sheep.GB: Gobi short tail sheep, UM: Ujimqin sheep, MG: Mongolia sheep. Indel-10-bp: g.30058882_30058873GCAGATTAAAIndel. Indel-3-bp: g.30483678_30483676AGAIndel. n.s.: Non-significant; *: $p < 0.05$; **: $p < 0.01$.
