# Supplementary material for: The Impact of Novel BMPR1B Mutations on Litter Size in Short-Tailed Gobi Sheep and Larger-Tailed Ujimqin Sheep
Source: Vet Sci. 2024 Jul 1;11(7):297. doi: 10.3390/vetsci11070297 (PMC11281403; doi:10.3390/vetsci11070297)
Supplement: Supplementary file 1 [file vetsci-11-00297-s001.zip › Table S1 MassARRAY primers used for genotyping of 10 variants in BMPR1B and 1 variant in T gene..pdf]

Table S1. MassARRAY primers used for genotyping of eleven variants in *BMPR1B* and one variant in *T* gene.

**Mutation**	**Primer Sequence (5'-3')**	**Annealing Temperature (°C)**
g.30483678_30483676 AGAIndel	F: ACGTTGGATGCACTCTGATGTAGTTTTCC<br>R: ACGTTGGATGATTTAAGAAAAGGGAGGAG<br>E: CTTAGCCTACCATTTT	51.6
c.30484046C>T	F: ACGTTGGATGTTCTACCTTGGGATTTTGGG<br>R: ACGTTGGATGTGTCATTGGCACCATCTCAC<br>E: GCTAGAATGTAGGTTG	52.4
c.684G>A	F: ACGTTGGATGCAATCTGTAGTGCCGTGAAC<br>R: ACGTTGGATGCCAATCTGTTTCACCATCTG<br>E: GGTTAACAGGTCCAGAG	52.4
g.30058882_30058873 GCAGATTAAAIndel	F: ACGTTGGATGTGCTTGCTAAACTCCATGTG<br>R: ACGTTGGATGAGTGCTTCGTGTTACTCAAG<br>E: GGAAGATGTGAGGTTCTTT	54.6
c.1560C>A	F: ACGTTGGATGAGCTTAATGTCCTGGGACTC<br>R: ACGTTGGATGAATCCTGCCTCAAGACTGAC<br>E: GGGGCTGACATTTTGGCAAG	55.0
c.1203A>C	F: ACGTTGGATGGCCTGACTTTCTCCTTAGTG<br>R: ACGTTGGATGACACTTCTGGAGGCATGTAG<br>E: TTGCCATACCACCCAACACTCG	62.4
c.687G>A	F: ACGTTGGATGCCAATCTGTTTCACCATCTG<br>R: ACGTTGGATGCAATCTGTAGTGCCGTGAAC<br>E: ACCCTCTGAATTTGCTTTGCTAT	57.4
g.30032124T>A	F: ACGTTGGATGAAACTTCTGTTTGTCCAGGC<br>R: ACGTTGGATGAAGATGTTCCAGCAGCATCC<br>E: CTTTTTGTCCAGGCTGCTCCCTGAG	71.4

g.30483701T>A	F: ACGTTGGATGTTTTCTTTTCACCTCCTCCC<br>R: ACGTTGGATGCTTGTAAGCTCAAAGGATTG<br>E: CCTCCTCCCTTTTCTTA	56.6
FecB	F: ACGTTGGATGCCAAGATGTTTTCATGCCTC<br>R: ACGTTGGATGTTCTTCACTACAGAGGAGGC<br>E: CCTCATCAACACCGTC	46.7
T Gene	F: ACGTTGGATGTGGATGTAGACGCAGCTGG<br>R: ACGTTGGATGACCGCTGGAAGTACGTGAAC<br>E: CCCCTGCTCTGGCTTGCCCC	65.2

Note: F: forward primer. R: reverse primer. E: extended primer.
