# Supplementary material for: The Impact of Novel BMPR1B Mutations on Litter Size in Short-Tailed Gobi Sheep and Larger-Tailed Ujimqin Sheep
Source: Vet Sci. 2024 Jul 1;11(7):297. doi: 10.3390/vetsci11070297 (PMC11281403; doi:10.3390/vetsci11070297)
Supplement: Supplementary file 1 [file vetsci-11-00297-s001.zip › Table S2 Genotypic, allelic frequencies and diversity parameters of 11 SNPs in the Gobi short tail sheep and Ujimqin sheep populations..pdf]

Table S2. Genotypic, allelic frequencies and diversity parameters of eleven SNPs in the Gobi short tail sheep and Ujimqin sheep populations.

Mutation/Breed	Genotype Frequency			Allele Frequency		Diversity Parameter				
						H_o	H_e	n_e	PIC	χ^2 (HWE[2])
c.684G>A	GG	GA	AA	G	A					
GB	0.810	0.186	0.004	0.903	0.097	0.824	0.176	1.213	0.160	0.795
UM	0.699	0.255	0.046	0.827	0.173	0.714	0.286	1.401	0.245	1.851
c.687G>A	GG	GA	AA	G	A					
GB	0.364	0.459	0.177	0.593	0.407	0.517	0.483	1.933	0.366	0.562
UM	0.340	0.484	0.176	0.582	0.418	0.513	0.487	1.948	0.368	0.006
c.1203A>C	AA	AC	CC	A	C					
GB	0.234	0.519	0.247	0.494	0.506	0.500	0.500	2.000	0.375	0.354
UM	0.118	0.373	0.510	0.304	0.696	0.577	0.423	1.733	0.334	2.185
c.1560C>A	CC	CA	AA	C	A					
GB	0.364	0.463	0.173	0.595	0.405	0.518	0.482	1.930	0.366	0.346
UM	0.288	0.516	0.196	0.546	0.454	0.504	0.496	1.983	0.373	0.262
g.30484046C>T	CC	CT	TT	C	T					
GB	0.779	0.203	0.017	0.881	0.119	0.790	0.210	1.265	0.188	0.208
UM	0.824	0.170	0.007	0.908	0.092	0.834	0.166	1.199	0.153	0.075
g.30483701T>A	TT	TA	AA	T	A					
GB	0.429	0.433	0.139	0.645	0.355	0.542	0.458	1.845	0.353	0.691
UM	0.294	0.451	0.255	0.520	0.480	0.501	0.499	1.997	0.375	1.429
g.30032124T>A	TT	TA	AA	T	A					
GB	0.671	0.286	0.043	0.814	0.186	0.506	0.494	1.976	0.372	1.530
UM	0.745	0.229	0.026	0.859	0.141	0.697	0.303	1.435	0.257	0.751
g.30483678_30483676 AGAIndel	AGA.AGA	AGA.DEL	DEL.DEL	AGA	DEL					

GB	0.528	0.372	0.100	0.714	0.286	0.501	0.499	1.997	0.375	1.429
UM	0.333	0.444	0.222	0.556	0.444	0.592	0.408	1.690	0.325	1.784
g.30058882_30058873 GCAGATTAAAIndel	GCAGATTAAA. GCAGATTAAA	GCAGA TTAAA.DEL	DEL.DEL	GCAGA TTAAA	DEL					
GB	0.658	0.290	0.052	0.803	0.197	0.684	0.316	1.463	0.266	1.597
UM	0.490	0.392	0.118	0.686	0.314	0.569	0.431	1.756	0.338	1.220
c.363G>T (*T Gene*)	GG	GT	TT	G	T					
GB	0.000	0.108	0.892	0.054	0.946	0.898	0.102	1.114	0.097	0.756
UM	0.949	0.051	0.000	0.974	0.026	0.950	0.050	1.053	0.049	0.095
c.746A>G (*FecB*)	AA	AG	GG	A	G					
GB	1.000	0.000	0.000	1.000	0.000	1.000	0.000	0.000	0.000	-
UM	1.000	0.000	0.000	1.000	0.000	1.000	0.000	0.000	0.000	-

Note: SE: Standard Error. H_o: Observed Heterozygosity. H_e: Expected Heterozygosity. n_e: Effective Allele Numbers. PIC: Polymorphism Information Content. HWE: Hardy-Weinberg Equilibrium. GB: Gobi short tail sheep. UM: Ujimqin sheep.
