# Supplementary material for: The Impact of Novel BMPR1B Mutations on Litter Size in Short-Tailed Gobi Sheep and Larger-Tailed Ujimqin Sheep
Source: Vet Sci. 2024 Jul 1;11(7):297. doi: 10.3390/vetsci11070297 (PMC11281403; doi:10.3390/vetsci11070297)
Supplement: Supplementary file 1 [file vetsci-11-00297-s001.zip › Table S3 Linkage disequilibrium as measured by D' and r2 among variants in the Gobi short tail sheep population.pdf]

Table S3. Linkage disequilibrium as measured by D' and r^2 among variants in the Gobi short tail sheep population.

	**g.30032124 T>A**	**c.1560 C>A**	**c.1203 A>C**	**c.684 G>A**	**c.687 G>A**	**Indel-10-bp**	**Indel-3-bp**	**g.30483701 T>A**
c.1560C>A	D' = 0.953<br>r^2 = 0.141							
c.1203A>C	D' = 1.000<br>r^2 = 0.235	D' = 0.211<br>r^2 = 0.030						
c.684G>A	D' = 0.730<br>r^2 = 0.013	D' = 0.703<br>r^2 = 0.036	D' = 1.000<br>r^2 = 0.105					
c.687G>A	D' = 0.124<br>r^2 = 0.005	D' = 0.291<br>r^2 = 0.084	D' = 0.073<br>r^2 = 0.004	D' = 1.000<br>r^2 = 0.074				
Insdel-10-bp	D' = 0.510<br>r^2 = 0.015	D' = 0.336<br>r^2 = 0.041	D' = 0.538<br>r^2 = 0.069	D' = 0.910<br>r^2 = 0.022	D' = 0.046<br>r^2 = 0.001			
Insdel-3-bp	D' = 0.015<br>r^2 = 0.000	D' = 0.043<br>r^2 = 0.000	D' = 0.135<br>r^2 = 0.008	D' = 0.132<br>r^2 = 0.005	D' = 0.018<br>r^2 = 0.000	D' = 0.117<br>r^2 = 0.001		
g.30483701T>A	D' = 0.142<br>r^2 = 0.003	D' = 0.107<br>r^2 = 0.009	D' = 0.074<br>r^2 = 0.003	D' = 0.175<br>r^2 = 0.002	D' = 0.039<br>r^2 = 0.001	D' = 0.329<br>r^2 = 0.015	D' = 0.196<br>r^2 = 0.028	
g.30484046C>T	D' = 1.000<br>r^2 = 0.031	D' = 0.331<br>r^2 = 0.022	D' = 0.522<br>r^2 = 0.036	D' = 0.436<br>r^2 = 0.003	D' = 0.290<br>r^2 = 0.017	D' = 0.040<br>r^2 = 0.000	D' = 0.010<br>r^2 = 0.000	D′ = 0.810<br>r^2 = 0.161

Note: Indel-10-bp: g.30058882_30058873GCAGATTAAAIndel. Insdel-3-bp: g.30483678_30483676AGAIndel.
