# Supplementary material for: The Impact of Novel BMPR1B Mutations on Litter Size in Short-Tailed Gobi Sheep and Larger-Tailed Ujimqin Sheep
Source: Vet Sci. 2024 Jul 1;11(7):297. doi: 10.3390/vetsci11070297 (PMC11281403; doi:10.3390/vetsci11070297)
Supplement: Supplementary file 1 [file vetsci-11-00297-s001.zip › Table S4 Linkage disequilibrium as measured by D' and r2 among variants in the Ujimqin sheep population.pdf]

**Table S4.** Linkage disequilibrium as measured by D' and $r^2$ among variants in the Ujimqin sheep population.

| | **g.30032124 T>A** | **c.1560 C>A** | **c.1203 A>C** | **c.684 G>A** | **c.687 G>A** | **Indel-10-bp** | **Indel-3-bp** | **g.30483701 T>A** |
|---|---|---|---|---|---|---|---|---|
| c.1560C>A | D' = 0.824<br>$r^2$ = 0.092 | | | | | | | |
| c.1203A>C | D' = 1.000<br>$r^2$ = 0.374 | D' = 0.442<br>$r^2$ = 0.071 | | | | | | |
| c.684G>A | D' = 1.000<br>$r^2$ = 0.034 | D' = 0.315<br>$r^2$ = 0.017 | D' = 1.000<br>$r^2$ = 0.091 | | | | | |
| c.687G>A | D' = 0.552<br>$r^2$ = 0.069 | D' = 0.331<br>$r^2$ = 0.095 | D' = 0.358<br>$r^2$ = 0.078 | D' = 1.000<br>$r^2$ = 0.151 | | | | |
| Indel-10-bp | D' = 0.111<br>$r^2$ = 0.004 | D' = 0.264<br>$r^2$ = 0.038 | D' = 0.241<br>$r^2$ = 0.012 | D' = 0.547<br>$r^2$ = 0.029 | D' = 0.161<br>$r^2$ = 0.017 | | | |
| Indel-3-bp | D' = 0.202<br>$r^2$ = 0.008 | D' = 0.005<br>$r^2$ = 0.000 | D' = 0.132<br>$r^2$ = 0.009 | D' = 0.405<br>$r^2$ = 0.027 | D' = 0.058<br>$r^2$ = 0.003 | D' = 0.027<br>$r^2$ = 0.000 | | |
| g.30483701T>A | D' = 0.285<br>$r^2$ = 0.014 | D' = 0.116<br>$r^2$ = 0.012 | D' = 0.152<br>$r^2$ = 0.009 | D' = 0.031<br>$r^2$ = 0.000 | D' = 0.063<br>$r^2$ = 0.003 | D' = 0.027<br>$r^2$ = 0.000 | D' = 0.053<br>$r^2$ = 0.002 | |
| g.30484046C>T | D' = 0.208<br>$r^2$ = 0.027 | D' = 0.030<br>$r^2$ = 0.000 | D' = 0.098<br>$r^2$ = 0.002 | D' = 1.000<br>$r^2$ = 0.021 | D' = 0.335<br>$r^2$ = 0.016 | D' = 0.030<br>$r^2$ = 0.000 | D' = 0.136<br>$r^2$ = 0.001 | D' = 0.720<br>$r^2$ = 0.056 |

Note: Indel-10-bp: g.30058882_30058873GCAGATTAAAIndel. Indel-3-bp: g.30483678_30483676AGAIndel.
